# Results of Modern Treatment of Pulmonary Consumption

**Published:** 1867-03

**Authors:** J. Henry Bennett


### Miscellaneous.

### Results of Modern Treatment of Pulmonary Consumption.

BY J. HENRY BENNETT, M. D.

*Acute Phthisis—General Phthisis—Chronic Phthisis—Phthisis among the Rich—Phthisis with Complications—Scrofulous Phthisis—Localized Phthisis—Phthisis of the Aged—Gouty Phthisis—Phthisis among the Poor.*

Having now briefly analysed the nature of pulmonary tuberculosis, and described its treatment by hygiene, climate, and medicine, I am desirous, in conclusion, to say a few words on the results obtainable, in accordance with my experience, by the employment of these means.

As I have already stated, by combining the various agencies which I have described, and which constitute what I have termed the modern treatment of phthisis, many patients may be and are saved; but many still die, and must ever die. The question I now purpose investigating, as far as possible, is: who are those who may live, and who are those whom all the resources of our art are unable to rescue from death? The answer to this question can only be approximated by referring to the laws of general pathology, by analysing the type of the disease, the circumstances under which it was generated, its stage of development when discovered and first treated, and its complications.

All diseases are greatly modified in their symptoms and progress, as also in the results of the treatment to which they are subjected, by the form or type which they assume from their first development, and none more so than pulmonary consumption. This disease may be acute or chronic.

In the acute type phthisis may run through all its stages in a few weeks or a few months. I have known patients seized with a series of febrile symptoms, having all the appearance of typhoid fever, and die in four or five weeks. On a post-mortem examination the lungs have been found full of miliary tubercles. No treatment has or can have any influence whatever on the termination of such a case; the patient is destined to die from the first day. In other equally fatal cases of acute phthisis, although the disease does not assume the form of a continued fever, and occupies several months, instead of several weeks, in passing through its successive stages, there is no lull whatever, no interval of arrest. The lung-tissue is progressively invaded by tubercular exudation, which rapidly softens, so that both lungs soon become a mass of advanced disease, and the patient dies without the disease having experienced any remission. What can medical science, climate, or hygiene do in such cases? Most surely is such a disease a mere mode of dying. Indeed, it is a question whether the most active and judicious treatment much retards the fatal issue.

Acute phthisis is much more frequently seen in youth than in middle or advanced age. The disease participates in the vigor and energy of the vital functions in early life. I consider it to be the evidence of a profound and final decay of vital power—so profound that there is no effort whatever made by the economy to contend with the evil that attacks it. The cause of acute phthisis must be sought for in the exaggeration of all the causes, hereditary and social, that produce the disease, and perhaps in their concentration in the same individual. Thus I have repeatedly witnessed it in persons who, with the hereditary predisposition or taint, have been exposed to extremely unfavorable hygienic conditions—town life, bad and scanty food, contaminated atmosphere, and great sorrows and cases.

Next in gravity to acute phthisis is the type of the disease in which the tubercular formation takes place, not in isolated patches, but all over both lungs simultaneously, at the apex, in the centre, and at the base. If the patient dies from other disease in the early stage of this form of phthisis, the lung is found studded with crude tubercles in its entire extent. When they soften simultaneously as they often do, the secondary bronchitis is generally severe, and the constitutional symptoms are very marked.

There are many degrees of intensity in this type of the disease; but the more the case recedes from tubercular development localized at the apex, the favorable type for treatment, the more serious is the prognosis.

When phthisis assumes the chronic type, as is generally the case, most fortunately, an unfavorable form for treatment is that in which disease shows itself in the midst of very favorable hygienic and social conditions. If a poor semptress, half starved, made to work eighteen hours out of the twenty-four, in a polluted atmosphere, living in a state of constant mental depression, becomes consumptive, common sense tells us that the disease may have manifested itself from the action of removable causes. If she can be placed under more satisfactory hygienic and social influences, she may therefore, and often does, recover. But if, on the contrary, the disease appears in one who has been bred and nurtured in the lap of luxury—who has known no hardship, no privation, no sorrow—of course the prognosis is more unfavorable.

It must be more difficult to arrest the progress of disease in such cases, for probably the cause is some strong hereditary predisposition, some defect originating with the progenitors, or some defective condition of individual innate vitality. It is in such cases, more especially, that everything should be done that is humanly feasible to arrest the disease, that no agency should be left untried that can possibly rouse the vitality of the patient. It is in such cases that he or she should be at once removed from the social medium in which the malady has been generated, in the hope of counteracting some unknown, unrecognized, and yet powerful home antagonistic influence. A change of climate is of inestimable value with these patients; indeed, it may be the only chance of arrest or recovery. Everything that is done, likewise, should be done from the very first; no time should be lost, for the foe is a most formidable one from the onset of the attack.

A class of cases still more inamenable to curative treatment is that in which there are serious complications present. Phthisis not unfrequently comes on in persons advancing in age, between thirty and sixty, who have led a hard life, who have taken large quantities of stimulants, and have exhausted perhaps an originally good constitution by excesses of various kinds. With them the stomach is generally out of order, the liver is often diseased, and sometimes the kidneys. What can treatment do in such cases? The disease may be considered a general break-up of the constitution, and the most judicious and persevering treatment seldom does more than retard the fatal termination.

Again, phthisis may attack at puberty those who during childhood have suffered from scrofula. This is a grievous and serious complication, but by no means so umpromising as those just described. Tuberculosis, or tubercular exudation, affects different organs at different periods of life. In infancy and early childhood it more especially attacks the meninges and the mesentery. In childhood and until puberty it attacks, in preference, the glandular structures of the neck, the extremities of the long bones, and the spongeous tissue of the bones in general, giving rise to the diseases of the articulations and bones which characterize scrofula. In early life, during my Paris career, I had charge for two years of a scrofulous ward of eighty young females, from fifteen to twenty years of age, in the hospital of St. Louis. They had nearly all glandular swellings, with or without scrofulous disease of the bones, ankles, knees, elbows, and a sad assemblage these poor girls were.

On several occasions I carefully examined the lungs of all my young patients, for I was publishing the clinical lectures of my master, the celebrated Dr. Lugol, and was much interested in everything connected with the pathology of scrofula. He wished to establish the connection betweeu scrofula and pulmonary consumption, and I found the evidence of localized tubercular deposits in the lungs of many of these crofulous girls. The tubercular lung deposits were met with mors especially amongst the elder ones, and that often although the patient presented little or no evidence of their presence. Dr. Lugol told me that he had long found this to be the case with his young scrofulous patients. When death, through accidental disease, afforded an opportunity for post-mortem investigation, the developmeut of tubercles in the lungs of scrofulous youths was much more frequently observed in those who were arriving or had arrived at puberty than those who were younger. Such tubercular exudations often remain crude and dormant for years, but when they assume a more rapid development and soften, the previous existence of scrofulous disease stamps the case as serious, although not necessarily as fatal. In these patients phthisis seems to appear, in its progressive form, as a species of climax to the antecedent tubercular or scrofulous affection. The crude tubercles which co-exist with scrofulous disease in the young, in the latent form, are no doubt often present without giving rise to any symptom, and they may be subsequently absorbed and the patient may recover without their presence having been even suspected.

The more favorable type of phthisis, that in which rational treatment is the most likely to arrest the progress of the disease, and even to effect a cure, is that which may be termed accidental phthisis, in a chronic form, localized to the upper region of the lungs. In this type of the malady there is no very decided hereditary taint, the patient is not born of very aged or very sickly parents, and does not present very serious complications in other organs, the evidence of a thorough and irremediable break up of constitution. Again, the disease generally manifests itself under the influence of overwork, sedentary town life, or harass, care, and anxiety. Sometimes in the most apparently luxurious and easy-going life some of these influences may be at work, so that appearances must not always be trusted. The habits and general life of persons moving in the highest circles, and having within their grasp every comfort, may be unhygienic. Moreover, they may be a prey, like humbler mortals, to cruel cares, none the less felt for not being recognized. Their nights may be sleepless, their days without joy; disappointed affections, social ties, or ambition may deprave digestion, and pave the way to the inroads of disease. In all such cases we may reasonably hope that the old saying, "Sublatâ causâ, attolitur effectus," may be verified. If we can remove *all* the causes that are depressing vitality, and the disease is in an early stage of its development, we may hope firstly to arrest its progress, and secondly to effect a cure, and that at any period of life short of extreme old age.

Pulmonary phthisis in extreme old age—not so rare an affection as is generally supposed—appears to me an all but incurable form of the disease, a mere mode of dying. I saw a number of cases of this form of consumption in the year 1840, when in medical charge of the infirmary of the Salpêtrière Hospital, Paris. This infirmary is fed by a population of 3,500 old infirm women, between 65 and 100, all living within the walls of this magnificent institution. The disease assumes the form of chronic bronchitis, but is characterized, besides the stethoscopic and percussion symptoms, by a most unearthly degree of emaciation. In no other disease have I seen patients live in such ghastly state of emaciation. They become at last like living mummies—nothing, literally, but skin, bone, and "concealed" organs.

There is a form of phthisis, of which I have seen a good many examples, although it is not generally pescribed. It may be termed gouty phthisis, and the prognosis, I consider, is rather favorable than otherwise, especially in its early stage. Consumption and gout are considered by many physicians to be antagonistic, but experience has proved to me that such is not the case; and the discrepancy between the theoretical and the practical view admits, I think of easy explanation.

Gout develops itself, primarily, in persons of healthy, robust constitution, who live generously. Their digestive system being good enables them to take and assimilate a considerable amount of nitrogenous food, and of stimulants, which appear to be the cause of gout developing itself. These people—the primarily gouty—do not become consumptive for their vitality is high and antagnistic to a disease of debility.

But when such robust gouty people who have themselves developed gout in their organization by a luxurious existence, marry late in life, as they often do, and have children, they do not generate children healthy and robust like themselves. Their children are often delicate, without being positively unhealthy; they have weak digestions, and suffer all their life from what may be termed gouty dyspepsia. If their organization is not much tried they get through life very well, and may reach old age, even when suffering more or less from the low forms of gout. If, on the contrary, they are much tried, body or mind—if they are placed for a continuance under unfavorable hygienic conditions, they fall below par, are liable to suffer from inflammatory affections of the aērial passages, which become chronic from lowness of general tone, and the deposit of pulmonary tubercle may follow. As I have stated, I do not think this form of phthisis an unfavorable one for treatment, for the constitution received from the parents is often originally a good one, merely weak and tainted with the low type of gout. There is often great latent vitality to work upon. My own case is one of this kind.

An all-important element in estimating the probable result of treatment—in forming a prognosis, in a word—is the extent of lung diseased when the patient is fairly brought under rational treatment. I often familiarly compare the lung attacked with tuberculosis to a large house on fire. The fire may begin in the servants' rooms or garrets—that is, at the top of the lung, the most frequent original seat of tubercular deposit. If it can be put out before it has extended to the story below, but little inconvenience is afterwards experienced by the owner of the house. He can live very comfortably in it under ordinary circumstances, only feeling that there is "less room" than formerly on extra occasions, such as visits from friends. If the story below, or the two stories below, are destroyed bofore the fire is put out, he feels more or less inconvenience in his "daily" life, but still he can get on. But when all the house is destroyed except one room, or the cellars, it becomes quite impossible for him to live in it by any amount of contrivance. Moreover, it is all but impossible to save even that one room when the fire has reached this point.

So it is with the lungs, which are not renewed, restored, when once destroyed; for we do not renew our organs as lobsters are said to renew their lost claws. Once a portion of the lung is destroyed, it is destroyed for ever, and its functions must be carried on by the healthy remainder. The only limit to curability, therefore, in my opinion, is the fact of there remaining a sufficient portion of healthy lung to carry on the functions of hæmatosis once the progress of the disease is arrested. The amount of healthy lung-tissue compatible with life evidently varies in different individuals according to their vitality. One lives long on a bit of healthy lung no larger than a small apple; another dies with even less than that in a state of disease.

The first and all-important point, therefore, is to arrest the progress of the disease, as it also is to arrest the fire in the house. Unless that can be done, in the one and the other case, the entire tenement will be destroyed, more or less rapidly. The living in the damaged tenement afterwards is a matter of adaptation; and it is wonderful what either nature or man can and will do to adapt themselves to altered circumstances. We must also bear in mind that the more the fire or the disease has progressed, when it is first discovered, the more difficult it always is to arrest it.

When, by the combined influence of hygiene, climate and medicine, the progress of phthisis has been arrested, crude tubercles have been absorbed or reduced to their mineral constituents, and cavities have been entirely, or all but entirely, cicatrized, it must not be supposed that the patient is well and safe. The recovery generally, always indeed, takes place through improved nutrition, and often the convalescent consumptive patient is fat and rosy, and looks healthy and well. But these looks are deceptive, the result of a life passed under the most hygienic circumstances possible, in unnatural quiet and repose. At the bottom there is still the tubercular cachexia, which reveals itself by a want of power, by lassitude, and even prostration, if the habits of invalidism are abandoned, and the sufferer once more quits the shores of the stream of life for the rapid current.

Consumptive convalescents should consider themselves invalids for years, and it is only by doing so that they can hope really to regain a firm footing in life. They may aptly compare themselves to a railway truck, "warranted to carry six tons," which after having been smashed, and then mended, painted and varnished, looks as good as new, but is not so. It may carry two or three, or even four tons safely, but no longer the original six, under penalty of a final catastrophe.

Those who cannot, or wlll not, thus consider themselves invalids, despite the outer appearance of health, relapse, and then all but invariably die miserably, for nothing saves them. I have now seen many such instances. One or two winters passed in the south, and rational treatment, arrest the disease, and bring, with the improvement, delusive confidence. The patient either cannot or will not listen to advice, and goes out again to fight "the battle of life," but only to relapse, and to return to the south in a hopeless condition.

What proves that even in those in whom the progress of pulmonary tuberculosis is arrested, tubercular cachexia, or defective vital power, long remains, is the frequency with which cachectic disease of another type subsequently attacks other organs. Thus last winter (1865–6) I lost at Mentone four patients from Bright's disease, all cases of arrested consumption. In one case consumption had been arrested for ten years, in another six, in a third two, in a fourth one. They all four died with all but complete lung quiescence; serious infiltration gradually rising until it reached the lungs, and then extinguishing life.

A year or two ago, some of the Parisian friends and companions of my younger days, now men of mature experience, and occupying the most prominent positions in the Parisian medical world, gave me a dinner as I passed through Paris on my way south. After we had dined, my case was talked over, and one after the other gave the results of his experience of the treatment of phthisis. All believed in its curability; all could quote cases of arrest and cure in their practice; but one and all stated that many of these cases of arrested consumption had subsequently died of some other form of cachectic disease, and principally from albuminuria, like my four patients of last winter.

Thus, perseverance and energy are long required, not only during the course of treatment, but for years after, if a thorough recovery is to be made, or even if a prolongation of enjoyable life is to be secured. This is really one of the most trying features of the disease, even when successfully treated. If we succeed in escaping death, we must accept invalidism for a long period, perhaps for the remainder of our lives. I would remark, however, that this applies more to the middle-aged who recover from phthisis than to the young. The latter have such an amount of organic activity about them, the characteristic of early life, that if they recover completely they may, with care, and by leading a hygienic life, regain a firm hold on life.

To secure this result I often advise my young made convalescent patients to abandon, if possible, sedentary pursuits, and to turn their thoughts to out-door occupations. Our Australian and South African colonies offer valuable fields for such persons. Life in the bush, among cattle and trees, in a dry climate like those I mention, is certainly more favorable to the prolongation of life in a tubercular convalescent patient than a city counting-house. Had I myself been a younger man, I should have adopted this course. As it is, I have come as near to it as possible by becoming an "amateur horticulturist."

Foolish people have scarcely a chance of recovery—they must perish. They generally do everything that is wrong and pernicious to please their own passing whims and fancies, and often look upon the friendly physician, who tries to rescue them from death, as one to be deceived and deluded. I repeat it. such unfortunate people have scarcely a chance of recovery. They have neither the sense to follow the right course when it is pointed out to them, or to grasp the hand of fellowship and sympathy when it is held out; nor will they sacrifice pleasure, money, or ambition to the pursuit of life. Indeed, I consider a weak, vacilating, peevish tone of mind, or an inordinate appreciation of, and clinging to, the enjoyments and possessions of life, to be as unfavorable an element of prognosis as any of those already discussed. Such mental conditions all but certainly preclude recovery, however favorable the case may otherwise be.

When I reflect on the convictions that have gradually gained ground in my mind respecting the treatment of phthisis—convictions embodied in this series of papers—I am often saddened by the thought, how are the poor to struggle successfully with such a disease? If rest from weary labors, if protection from atmospheric vicissitudes, if ample, nay, a luxurious dietary, if expensive medicines, such as cod-liver oil, if change of climate, to escape winter cold and wet, are necessary, how can those who live by their daily labor—and even many above them in social rank—hope to escape from the grasp of this fell disease? Is not the battle itself for them a hopeless one?

To these questions I would answer, that, although the struggle for life cannot, most assuredly, be made with the same chance of success by the poor as by those whose position enables them to do all that is calculated to arrest disease, yet their case is by no means a hopeless one. The means of treatment that I have recommended—hygiene, climate, medicine—may be attended to at home, in our own country, in the midst of the duties and occupations of life, although in a minor degree. I have met with cases of arrested and cured phthisis in persons who have never left England, and who have never given up their social pursuits; and so have other physicians. Some of the most satisfactory and conclusive cases given in Professor Bennett's valuable work "On Pulmonary Consumption," are cases of this description.

To attain this end, however, nothing should be neglected. Unhygienic, unhealthy occupation should be given up; all the rules of hygiene to which I have alluded should be scrupulously followed; out-door occupations substituted for in-door during the summer months, if possible; and, more especially, town should be abandoned for the country as a residence, whenever feasible.

Cities exercise a mysterious attraction over the lower as well as the higher classes of mankind. It must be the feverish excitement of city life, the hope of greater social advancement; for the greater portion of the lower classes in cities live as hard or harder lives than they would if similarly engaged in the country. No doubt the vitiated air breathed in cities, in the close crowded workshops, and in the closer and still more crowded sleepingrooms, gradually weakens the constitutional powers, and forms the principal predisposing cause of phthisis. The poor should return to their native villages, if by any means feasible, even if there they have to accept a lowlier position than that which they have attained. The younger members of the family, when attacked with phthisis, should be sent to board or work with country relatives. The country air would do them more good than all the physic they can get from hospitals and dispensaries in town, and give them a better chance of recovery. Indeed, it has often struck me that the funds of our city charitable institutions would be best employed by boarding their consumptive patients in farm-houses and agricultural villages, than in maintaining them in the wards of a city hospital. Or the hospital itself might be placed on some heathy, pine-covered moor, like the Convalescent Hospital at Walton-on-Thames; and out-patients only seen in town.

It must be well understood that I am now speaking only of *curative* treatment. If all hope of recovery has been abandoned, if the lungs are all but destroyed, and the disease can not be arrested—if an asylum to die in is all that is required—then it is of but little avail to drive the poor patient into the country, away from home ties and home sssistance. Then, when the last scene is at hand, any asylum will do to die in—the small home, with dear friends around, the city hospital, the workhouse infirmary.

The recent researches regarding nutrition, to which I have elsewhere alluded, are consolatory as regards the poor. As long as we believed that, in the scheme of nutrition, meat meant muscle and strength, fats and cereals heat only, the poor at home seemed to have but a slight chance of recovery in asthenic diseases, diseases of debility. With meat nearly a shilling a pound, how can they obtain ten shillings' worth each week? and if it is indispensable, how are they to get well without? But if, as is now stated—and, I believe, with truth—meat is principally a muscle-repairer, and the force created is in reality chiefly obtained out of the carbonaceous food, fats, and amylaceous substances, the chance of the poor is infinitely greater when "force" has to be regained. Oatmeal, or any cereal, with milk and oil or fat, in that case, do as well as butcher's meat, and a few shillings a week will go as far as ten.

I have certainly, throughout my professional career, remarked, as already observed, that the meat-fed children, and great meat-eaters, are not stronger than other people. With children, indeed, I believe it is the reverse. The children whom I have attended, who have lived on meat, eating it three times a day—certainly not by my advice—have not proved as strong nor as healthy as those who have lived on a more mixed dietary. Compare these town-fed children, who eat from ten to twenty shillings' worth of meat every week, with the Irish or Scotch peasant children, fed all but entirely on potatoes and milk, or oatmeal and milk. These researches also explain the disastrous effects which have, in many instances, resulted from the very nitrogenous or animalized dietary recently vaunted as a remedy for obesity.

Of course, I am well aware that the advice I now give can only be partially followed, that there ever will be persons affected with phthisis in all classes of society, by whom it must be accepted as the decree of Providence, and who must struggle with it *in situ.* But even in such cases, in the earlier stages of the disease, a curative treatment may be attempted by all, even those whose means are small, or who depend on their daily labor for their bread. In more advanced disease, likewise, a lull may be taken advantage of to make the attempt. Pulmonary consumption does not usually progress steadily, uninterruptedly; its very nature is, on the contrary, to advance *per saltum*—by jerks, as it were. When not treated, it generally remains stationary for a time; then progresses, then again remains stationary, to again progress. We may take advantages of these lulls, which represent Nature's own unaided efforts to limit and control the morbid action, in order to further hygienic treatment.

Following out this train of argument, I advise the young clerk, if able, as soon as a lull takes place, or is obtained by treatment, to give up sedentary pursuits, and turn farmer at home, in Australia, New Zealand, or the Cape. I advise the young artisan to abandon the town, and to follow his calling in the country. I advise the maid-servant or sempstress to leave the city, and to find service or work in some country place. Nearly all have country friends, who will help them in their efforts.

There was a time when, like my neighbors, in such cases, among the poor, I prescribed tonics, cod-liver oil, and a generous dietary, and thought my duty performed. Now I have learned better; I have learned to place little confidence in the curative value of mere medicinal treatment, pursued for a time, then abandoned. If the patients, whatever their class of life, remain exposed to the influences under which the disease is generated, their fate is generally sealed, whatever the treatment. Now, therefore, I try in such cases to encourage them to make family and social sacrifices which a more radical treatment of their disease entails. Family and social ties are as strong with the poor as with the rich; and the tendency is even stronger with them than with the better educated, to demand from the physician a remedy which is to cure their complaint without any change or sacrifice on their part. As I have repeatedly said in the course of this essay, no such remedy exists for pulmonary consumption, nor is it probable that it will ever be discovered.

The various cures for pulmonary consumption that are constantly brought forward, are founded on entire ignorance of the laws of general pathology. Those who are acquainted with these laws know well how utterly impossible it is for any one of the remedies proposed—for the inhalation of any medicinal substance, or of any amount of compressed air, or for any degree of forced inspiration—to cure a disease such as I have described, one of defective lowered vitality.

Nothing but an appeal to the laws that regulate the preservation and development of life can have that result. An intelligent application of those laws, as demonstrated by physiology, with the assistance of climate and rational therapeutics, may, however, be made most unquestionably the means of saving very many lives.

As I have stated in my work on Climate ("Winter in the South of Europe," 3d edition,) I am now surrounded, both at home and abroad, by a little tribe of friends and patients whose lives have been saved, like my own, by the steady application of these principles.—*London Lancet.*

